# High-Frequency Currents and the Treatment of Sciatica

**Published:** 1904-11-12

**Authors:** W. F. Somerville

**Affiliations:** Glasgow


### EDITOR'S LETTER-BOX.

[Our Correspondents are reminded that prolixity is a great bar to publication, and that brevity of style and conciseness of statement greatly facilitate early insertion.]

### HIGH-FREQUENCY CURRENTS AND THE TREATMENT OF SCIATICA.

*To the Editor of* The Hospital.

Sir,—In the issue of The Hospital of date October 22nd, a short article appears summarising some recent suggestions on the treatment of sciatica. You are kind enough in this to mention my name in connection with the treatment by high-frequency currents. May I venture to suggest to those medical brethren who are uncertain whether or not to employ this form of treatment in cases of sciatica to make up their minds whether the patient in whom they are interested is suffering from a simple neuralgia of the sciatic nerve or from a perineuritis with resulting adhesions between the nerve and its sheath? In the former case the pain complained of is felt while the patient is at rest as well as when he indulges in active movement. It is specially severe in the morning when the patient rises; as the day advances, however, the movement of the limb causes a lessening of the pain. On the other hand, in those cases where there is a perineuritis, little or no pain is complained of while the patient is at rest, but as soon as he moves the limb, pain varying in intensity, and caused by the adhesions dragging on the nerve tissue is experienced. Only those who have seen a surgeon separating these adhesions can fully appreciate their extent and possibilities. In the neuralgic cases, further, it may be noted that the small sciatic nerve on the same side is frequently involved, and pain is complained of over the gluteal region. It is in the cases of neuralgia pure and simple that one may confidently expect speedy and marked relief by means of the high frequency currents. On the other hand, it is beyond the possibility and province of these currents to diminish or remove pains that are caused by gross pathological changes and such mechanical interference as results from the existence of perineuritic adhesions.

Yours faithfully,

Glasgow. W. F. Somerville, M.D.

